# Circadian rhythms in pediatric neurocritical care: interfaces and opportunities

**DOI:** 10.3389/fneur.2026.1826615

**Published:** 2026-07-30

**Authors:** W. Michael Babinchak, Jonathan O. Lipton

**Affiliations:** 1 Department of Neurology, Boston Children’s Hospital, Boston, MA, United States; 2 F.M. Kirby Neurobiology Center, Boston Children’s Hospital, Boston, MA, United States; 3 Division of Sleep Medicine, Harvard Medical School, Boston, MA, United States

**Keywords:** child neurology, circadian rhythms, intensive care unit, neurocritical care, sleep disturbance

## Abstract

Neurological disorders represent a disproportionate fraction of pediatric intensive care unit (PICU) admissions, accounting for approximately one quarter of all critically ill children. These conditions—including traumatic brain injury, hypoxic–ischemic encephalopathy, stroke, infectious and inflammatory disorders, epilepsy, and disorders of consciousness—are frequently accompanied by profound disruption of circadian rhythms. The circadian timekeeper is a fundamental biological system that coordinates physiology across scales, from molecular and cellular processes to systemic functions such as metabolism, immunity, thermoregulation, endocrine signaling, vascular regulation, synaptic physiology, and sleep–wake cycling. In the PICU and neonatal ICU (NICU) environments, multiple factors—including continuous or irregular lighting, noise, frequent clinical interventions, sedation, altered feeding schedules, and the pathophysiology of neurological injury itself—conspire to destabilize circadian organization. We summarize these contributing factors into pathways of critical care circadian desynchrony (C3D). This review examines the intersection between circadian biology and pediatric neurocritical care. We first summarize the molecular and systems-level architecture of the circadian system and distinguish circadian rhythms from sleep–wake state regulation. We then outline the development of sleep and circadian rhythms in the fetus, neonate, infant and child, emphasizing that pediatric circadian biology cannot be treated as a simple extension of adult neurology and chronobiology. We consider how the PICU and NICU environments disrupt circadian biology and discuss how circadian dysregulation manifests across common neurological conditions encountered in pediatric critical care including hypoxic–ischemic encephalopathy (HIE), traumatic brain injury (TBI), stroke, infection and autoimmune encephalitides, epilepsy, delirium, and status dystonicus. Emerging literature suggests that disturbances of circadian outputs—including hormone secretion, metabolism, immune signaling, thermoregulation, sleep–wake cycling, autonomic regulation, and gene expression—may influence neurological injury progression, recovery trajectories, and long-term neurodevelopmental outcomes. We review current methods used to measure sleep and circadian rhythms in critically ill children, including polysomnography, electroencephalography, actigraphy, biomarker-based approaches, and emerging physiological metrics derived from continuous monitoring. Finally, we discuss potential clinical strategies aimed at restoring circadian alignment in the PICU environment, including environmental modification, sedation-aware care, pharmacologic chronobiotics, time-structured nutrition, and chronotherapy. Together, we offer a conceptual framework that centralizes circadian desynchronization in pediatric neurocritical care as an under-explored, potentially targetable modifier of neurological injury and recovery in pediatric critical illness. Integrating circadian biology into pediatric neurocritical care may provide new opportunities for biomarker development, therapeutic intervention, prognostication, and personalized chronomedicine.

## Introduction

Neurological disorders comprise many of the admissions to the pediatric intensive care unit (PICU). Whereas these disorders make up about 10.7% of overall hospitalizations, they disproportionally represent 23–25.8% of PICU admissions (1, 2). This highlights the importance of understanding fundamental mechanisms that contribute to pediatric neurological disease in critical care medicine. The most frequently encountered neurological disorders that require critical care are traumatic brain injury (TBI, comprising 55%), neuro-infectious and inflammatory diseases (16%), status epilepticus (14%), stroke (9%), hypoxic ischemic injury (5%), and spinal cord injury (2%) (1).

Life on a rotating planet exposes humans of all ages to predictable 24-h light/dark cycles. To synchronize with this predictable environmental oscillation, the circadian timekeeping system has evolved to anticipate time of day across biological scale—from protein function and cellular metabolism to tissue coordination and complex behaviors such as sleep–wake cycling and cognition (3–5). Misalignment between the individual and the environment, or between tissues *within* an individual, results in circadian desynchrony. Disrupted circadian rhythms have been associated with irregular sleep/wake cycles, metabolic derangement, inflammation, and hormonal imbalance, altered thermoregulation, immune dysfunction, and impaired cognition.

Circadian rhythm disruption in children cannot be understood in isolation from the developmental and chronological context of the individual child. Circadian rhythm disruption in pediatric neurocritical illness should be interpreted in the context of gestational age, postnatal age, neurological maturation, injury state, medication exposure, and environmental conditions. The circadian timekeeping system functions as an allostatic organizer that coordinates and rebalances physiology, gene expression, cellular function, and behavior (6–9). This is particularly important in pediatrics because circadian biology changes dramatically across development. Most research on circadian rhythms in humans has been conducted in adults or animal models, yet the interface between daily circadian rhythms and developmental stage is likely critical for measuring, understanding, and mitigating circadian disruption in pediatric neurocritical care. While circadian rhythms change across the lifespan, how the interactions between changes in daily rhythmicity and developmental trajectory is altered by critical care remains unknown.

Circadian rhythms are adversely affected by the ICU environment, the complexities of acute care, and the underlying processes that result in admission to a critical care unit. Within the ICU, fragmented light/dark cycles, excessive or continuous light exposure, noise, altered nutrient timing, frequent shift changes, physical disturbances by caregivers and staff, and pharmacological sedation cumulatively destabilize sleep and circadian organization. Evidence from adult critical care studies suggests that disruption in the duration, architecture, and quality of sleep and circadian rhythms commonly occurs in the ICU and is associated with increased mortality and extended length of stay (10). Despite these supportive studies in adult critical care medicine, studies of circadian rhythm dysregulation in the PICU still remain sparse. The available pediatric studies demonstrate disrupted sleep architecture, including reduction in rapid eye movement (REM) sleep (11), minimal or absent N3 sleep (12, 13), and circadian dysregulation (11) in the PICU. These disturbances may continue to impact on health-related quality of life in children even after discharge from the PICU (14).

The neurological care of children cannot be transferred in modular fashion from evidence obtained in adults. Children differ from adults in sleep architecture, circadian consolidation, developmental neurobiology, metabolic physiology, immune development, pharmacology and mechanism of neurological disease. This review therefore examines circadian disruption in pediatric neurocritical care uniquely through developmental and translational perspectives. We first review the circadian system and its major outputs, then consider the developmental emergence of sleep and circadian rhythms. We next examine the PICU and NICU as circadian-disruptive environments. We then review common pediatric neurocritical care conditions through the lens of circadian biology, followed by current measurement strategies and emerging opportunities for pediatric circadian medicine.

## The circadian timekeeping system across biological scales

### Multiscaled temporal organization

**Figure 1:**
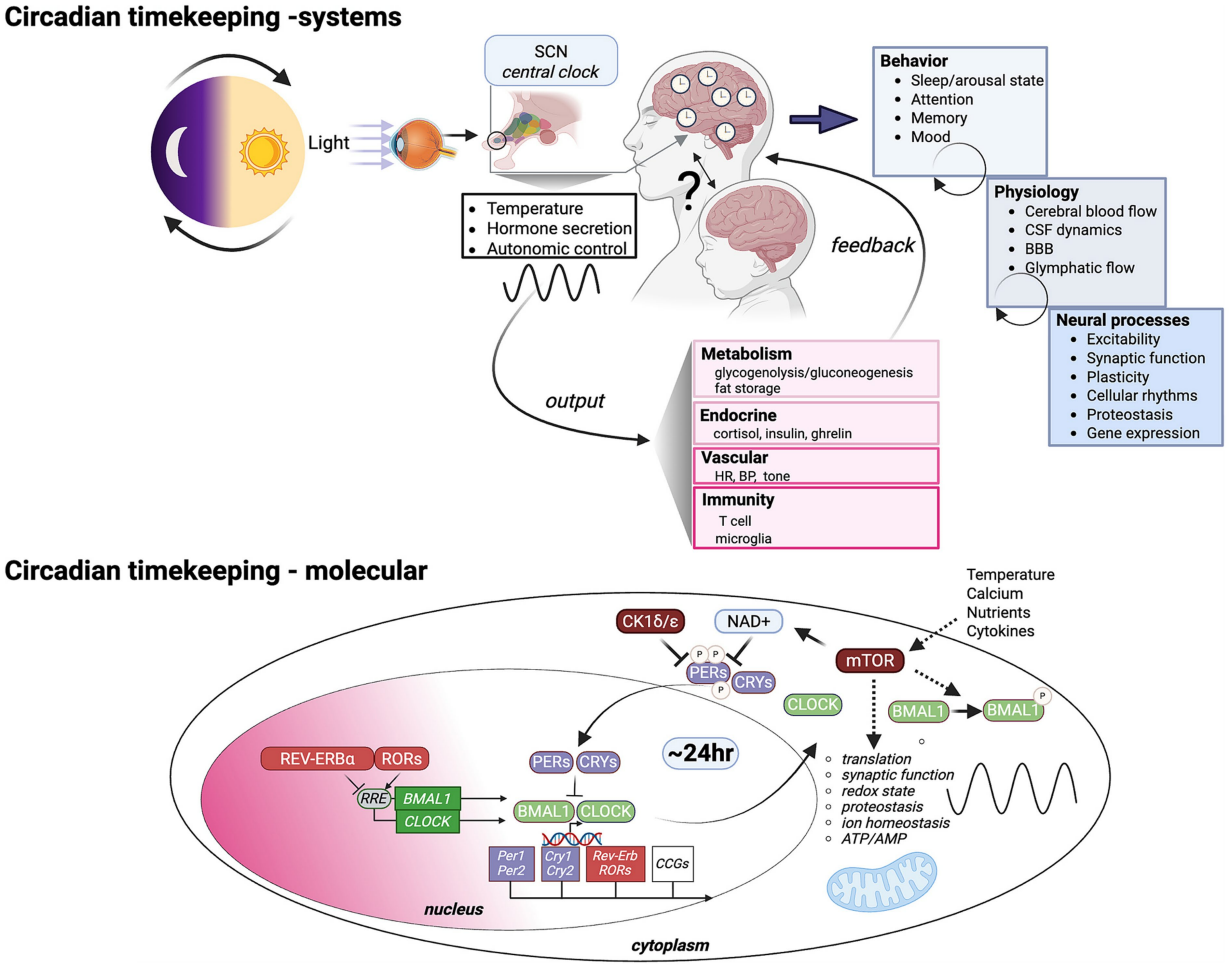
(Top) Circadian timekeeping at systems and molecular levels. The suprachiasmatic nucleus (SCN) of the hypothalamus is the central clock. Light is received via the retinohypothalamic tract and conveyed to the neural network within the SCN, which synchronizes neuronal activity with oscillation of the molecular clock (Bottom). The SCN has widespread neural and humoral interactions within the hypothalamus and brainstem, organizing rhythms of temperature, hormonal secretion, and sympathetic and parasympathetic function. Circadian clocks are present in virtually all tissues including the liver, bone marrow, fat, adrenals, lungs, and heart. Major outputs include circadian oscillations in glucose metabolism, mitochondrial function, vascular tone, immune cell regulation, and hormonal systems. Many of these feedback on clocks in the brain and possibly the SCN (although the SCN is impervious to temperature rhythms even though it regulates them). In the brain, clocks are presented here across scale, ranging from complex behaviors like sleep/wake timing, to mood regulation. Circadian rhythms of blood flow, the opening/closing of the blood–brain barrier (BBB), CSF flow and molecular dynamics, and glymphatic clearance compartments are presumably coordinated; however, much more work is needed. The clock also regulates synaptic function, excitability, ion flux and is expressed at the molecular level in neurons, glia, and brain immune cells. The molecular clock is a conserved transcription-translation feedback loop that is partially schematized here. BMAL1 and CLOCK form dimers that seed a multiprotein complex that engages the promoters of many thousands of genes including the *PERIOD* (*PER*) and *CRYPTOCHROME* (*CRY*), *REV-ERBα,* and many ‘clock-controlled genes’ (CCGs). PER1/2 and CRY1/2 proteins undergo post-translation modification and upon shuttling back to the nucleus, inhibit the BMAL1/CLOCK complex. Degradation of PER and CRYs then release BMAL1/CLOCK to renew another cycle, completing the loop. In the cytoplasm, circadian clock machinery intersects with major cellular signaling pathways including the mechanistic target of rapamycin, SIRT1, and Casein kinase 1d and e, among many others. While not shown, the proteins undergo tightly regulated targeted degradation that is thought to contribute to the timekeeper. Finally, clock proteins also have biochemical functions in the cytoplasm. For example, BMAL1 ‘moonlights’ as a promoter of translation and a modulator of synaptic function via biochemical regulation of synaptic proteins such as CaMKIIα. The molecular clock therefore is integrated with core cellular machinery thought to mediate metabolic and physiological homeostasis.

The light/dark cycle imposes an environmental pattern on the availability and utilization of energy. As a result, organisms across kingdoms of life have evolved a multi-scaled biological timekeeping system – the circadian clock – that anticipates this environmental variability by transforming it into a ~ 24-h (“circa” + “diem,” about a day) rhythm of gene expression, proteostasis, metabolism and physiology. This timekeeping system organizes daily rhythms in temperature, hormones, synaptic function, immune signaling, metabolism, and behaviors such as sleep/wake cycles, attention, memory, and mood (15, 16) (Figure 1).

### The SCN

The mammalian circadian system is hierarchically organized around a central timekeeping mechanism in the suprachiasmatic nucleus of the hypothalamus (SCN). The SCN maintains a roughly 24-h periodicity through coupled rhythmic gene expression, neuronal activity, and astrocytic buffering (Figure 1). It transmits timing cues through both neural, humoral signals, and autonomic, and temperature-related pathways. The SCN integrates photic and non-photic afferent information to regulate sleep–wake state and physiological timing (4). Photic input begins with melanopsin-containing, non-image-forming retinal ganglion cells that project through the retinohypothalamic tract to the SCN. Humoral inputs, including melatonin, androgen, estrogen, and progesterone, can also regulate the SCN.

SCN efferent pathways involve neural connection with hypothalamic and brain stem nuclei, including the dorsomedial hypothalamus (DMH), paraventricular nucleus, preoptic area, locus coeruleus (LC), and serotonergic raphe nuclei. These brainstem and hypothalamic nuclei work in concert to regulate sleep and wakefulness, temperature fluctuations, hormonal pathway, arousal states and autonomic rhythms (5, 17, 18). Sleep-promoting neurons in the ventrolateral preoptic area and wake-promoting neurons in the lateral hypothalamus are directly influenced by the DMH, which receives inputs form the SCN. The LC further facilitates arousal and sleep-state transitions via input from these hypothalamic circuits. Thus, the SCN is positioned atop a complex neural network that regulates the timing of transitions between global brain states and rhythmic physiological outputs conveyed to the body (Figure 1).

### The molecular clock

At the cellular level, circadian rhythms are generated by a ubiquitous molecular clock mechanism (Figure 1). The transcription factors BMAL1 and CLOCK form a dimer that seeds a multiprotein assembly that regulates many thousands of genes including those that ultimately inhibit them – the Period and Cryptochromes (19). In addition to this primary loop, various accessory loops, including those mediated by the transcriptional regulator REV-ERBα and RORs, provide additional levels of robustness to the timing mechanism. Phosphorylation by cytoplasmic kinases regulates the stability and nuclear shuttling of the clock machinery, thereby facilitating temporally-controlled degradation of PER/CRY inhibition, allowing the cycle to re-initiate (20). Together, this network is referred to as the transcription-translation feedback loop (TTFL).

The TTFL regulates the rhythmic output of thousands of genes, many with tissue- or cell-type specific rhythms, phases, and amplitudes. About 70% of synaptic genes are under circadian clock control in the forebrain (21). Crucially, cellular rhythms extend beyond mRNA rhythms to include protein synthesis and degradation (22), mitochondrial function (23), ion flux (24), ribosome biogenesis (25), and, in neurons, synaptic function (26–28). Thus, circadian timekeeping is not merely a sleep–wake system; it is a systems-level architecture for coordinating metabolism, cellular physiology, excitability, immune function, vascular behavior and neurobiological state.

The phase of the circadian timekeeper shifts in response to environmental cues (i.e., zeitgebers), such as light, nutrients, temperature, melatonin, and redox state with phase-dependent specificity (19, 29, 30)(Figure 1). Of these, light is the most potent, reaching the SCN via the non-image forming retinal ganglion cells and the retinohypothalamic tract (Figure 1). Other zeitgebers may act on peripheral clocks, the central clock, or both. Individual sensitivity to zeitgebers may contribute to variability in circadian timekeeping.

## Sleep, circadian rhythms, and development

Sleep–wake behavior is perhaps the best recognized output of the circadian timekeeper and is often confused with circadian rhythmicity itself. Although the circadian system regulates the timing and architecture of sleep cycles, strictly clock-controlled processes are those that depend primarily on intrinsic circadian state, or phase, rather than behavioral or physiological history. Melatonin secretion is a canonical example. By contrast, arousal state exerts state-dependent influence on many physiological parameters, which may or may not align with intrinsic circadian phase (31). Growth hormone secretion, for example, depends strongly on slow-wave sleep rather than simply on circadian night (32, 33).

Sleep is regulated by the combined influence of the circadian clock, which determines the timing of sleep–wake transitions and contributes to sleep architecture, and a homeostatic mechanism, which regulates sleep drive as a function of prior wakefulness. Although the classical two-process model distinguishes a state-independent clock from a state-dependent homeostat, these processes interact substantially (18). Many physiological variables, including temperature and heart rate, are influenced by both circadian phase and arousal state. Alterations in sleep–wake cycling can influence circadian gene expression, and alterations in circadian genes can dysregulate sleep homeostasis in humans and animal models. A major challenge in pediatric neurocritical care is therefore to distinguish circadian disruption from factors that alter behavioral state, arousal, or sleep architecture—or to determine when both are affected simultaneously.

**Figure 2:**
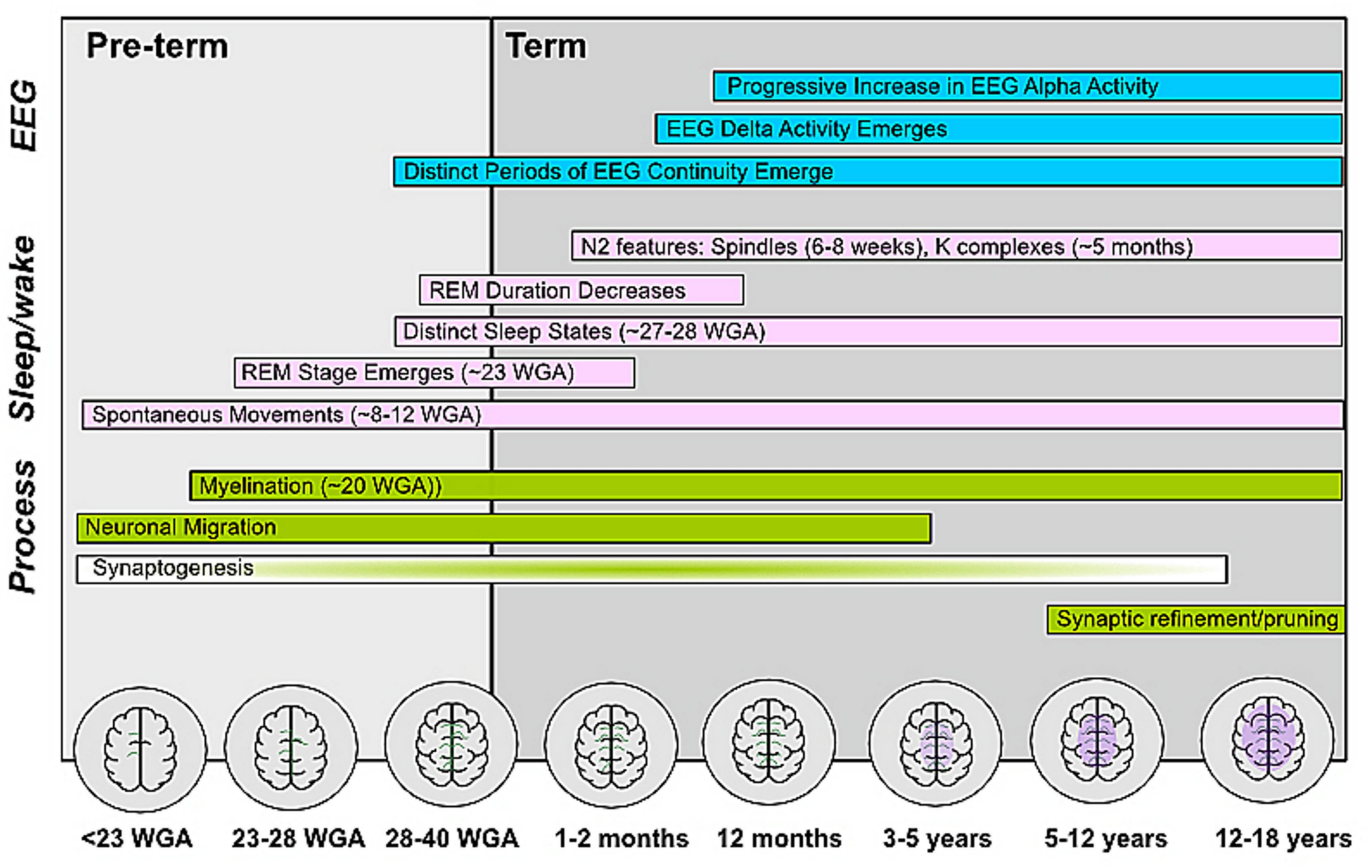
Ontogeny of human sleep, EEG findings, and brain developmental milestones. While it is not known exactly when the human circadian oscillator begins cycling, animal models suggest that the SCN is rhythmic during the analog of the third trimester in humans. Infants usually demonstrate ultradian rhythms of behavior and sleep cycling rapidly between REM- and NREM-like states. Consolidated circadian rhythms of sleep/wake and feeding behaviors start around 5 months. Sleep is highly developmentally regulated in a manner that broadly overlaps in the posterior–anterior progress of myelination and the transition from synaptogenesis in young children to synaptic refinement and pruning in adolescence that correlates with the onset of adult-pattern sleep architecture. Causal relationships between these associated milestones have not been experimentally determined.

Most adults are diurnal: behavior comprises a single consolidated wake state and a single consolidated sleep state (18). Sleep is divided into rapid-eye movement (REM) sleep and non-rapid-eye movement (NREM) sleep. REM sleep is characterized by muscle atonia, rapid eye movements, and phasic periods of poikilothermy. NREM sleep is divided into N1, N2, and N3 stages, which reflect increasing sleep depth. N1 is characterized by slower frequencies on electroencephalography (EEG) and suppression in background EEG amplitude. N2 is characterized by sleep spindles and K complexes. Slow-wave sleep predominates the N3 stage (Figure 2). Sleep architecture, especially the distribution of REM sleep, is strongly ensconced by the circadian system.

The emergence of the circadian rhythm and sleep architecture involves developmental stages that begin in the fetal stage and continue through the post-natal period. Spontaneous movements can be observed as early as 8–12 weeks gestational age, whereas a distinct REM stage is not clearly evident until around 23 weeks (34). Around 27–28 weeks, distinct sleep states begin to emerge, including quiet sleep and active sleep, which correspond with NREM and REM-like states, respectively (35). Around this time, continuous EEG patterns emerge from a primarily discontinuous pattern (36). By 30 weeks, most sleep is spent in a REM-like state, which gradually decreases from 80% at 30 weeks to 58% by 36–38 weeks (35). An ultradian rhythm cycling every 70–90 min begins around 32 weeks (37).

Sleep architecture undergoes massive reorganization during the first year of life (Figure 2). Newborns cycle frequently between active REM-like sleep and quiet NREM-like sleep. Active sleep can comprise more than 50% of sleep architecture and transitions between active sleep, quiet sleep, and wakefulness are frequent (38). The duration of each REM cycle is longest immediately after birth and estimated to be about 30 min in infants aged 2–4 weeks (39), shortening to 16 min by 16–24 weeks (39). By 3 months of age, REM sleep begins to encompass a greater percentage per hour of sleep during the night than during the day (40). Quiet sleep also undergoes changes in the first weeks of life: mature spindles appear between 6 and 9 weeks, slow delta waves mixed with theta frequencies emerge around 12 weeks, and the number of K complexes continues to increase through 6 months (36, 38, 40).

The intersection of maternal-fetal rhythms is a growing area of investigation, primarily supported by rodent studies. From the fetal perspective, the murine SCN can develop intrinsic rhythmicity even when explanted from surrounding embryonic cells at embryonic day 14 (41). Synchronization between maternal and fetal rhythms occurs early and almost certainly during the latter third of gestation (41, 42). Cycling of glucose utilization in fetal rats reflects the sleep–wake cycling of their mothers as early as embryonic day 19 (43).

Neurohumoral mediators, including melatonin, are thought to facilitate maternal-fetal synchronization (44). Melatonin administration to term women has been reported to result in increased serum melatonin levels within the umbilical circulation and may entrain the fetus (45, 46). Other proposed mediators include dopamine, which may also undergo rhythmic fluctuations with the potential for crossing the placenta, and corticosterone, which crosses the placenta and is important for fetal organ maturation (44). Disruption of maternal circadian rhythms during pregnancy in mice has been shown to predispose offspring to diet-induced obesity, whereas reestablishing these rhythms rescued the phenotype (47). Whether similar mechanisms apply in human gestation remains to be determined.

## Major outputs of the circadian system relevant to neurocritical care

In addition to sleep, the circadian system has many physiological outputs (19, 48). While a comprehensive review of each output is outside the scope of this review, it is helpful to briefly conceptualize the impact of circadian dysregulation within the critical care setting. For this discussion, the most relevant circadian system-regulated outputs include metabolism, temperature patterns, immune system activity, and neuronal and synaptic biology.

Many metabolic pathways demonstrate circadian behaviors, ranging from insulin release to glucose and fatty acid metabolism, cholesterol metabolism, heme regulation, and NAD production (49, 50) (Figure 1). These rhythms are driven by autonomous clocks in resident tissues, such as the liver and pancreas, while also being influenced by the SCN. Circadian fluctuations in RNA and protein expression contribute to rhythmic metabolic function as well.

Temperature regulation also reflects the resilience and adaptability of the circadian system. Environmental temperature can act as a zeitgeber, particularly for peripheral clocks, but the SCN itself is relatively resistant to temperature entrainment. Thus, body temperature is primarily an output of the central clock and only weakly an input to it. This property allows the SCN to orchestrate rhythmic fluctuations in body temperature without being constantly reset by environmental temperature. Thermoregulatory precision is relevant to brain temperature, cerebral blood flow, infectious responses, and metabolic cycling (4, 18, 51).

The immune system is also regulated by the circadian clock. Rhythmic fluctuations in monocytes and macrophages influence responsiveness to bacterial antigens and fluctuations in cytolytic proteins produced by natural killer (NK) cells (52). Circadian fluctuations in lymphocyte population number, expression of clock genes within lymphocytes, and tissue-specific recruitment are also seen. This periodicity is proposed to play a role in the daily pattern of symptoms seen in asthma as well. The complex interaction between sleep, circadian rhythms, and the immune system are thought to play important roles in the pathogenesis of neuroimmunological disorders (53).

Circadian rhythms also regulate neuronal and synaptic biology. Clock-regulated transcription influences synaptic gene expression, protein translation, mitochondrial function, ribosomal biogenesis, ion homeostasis, and synaptic plasticity (5, 21, 22, 25, 28, 54–59). These pathways are directly relevant to excitability, seizures, neurodevelopment, recovery after injury, and neurocritical care outcomes.

## The critical care unit environment as a circadian perturbation

**Figure 3:**
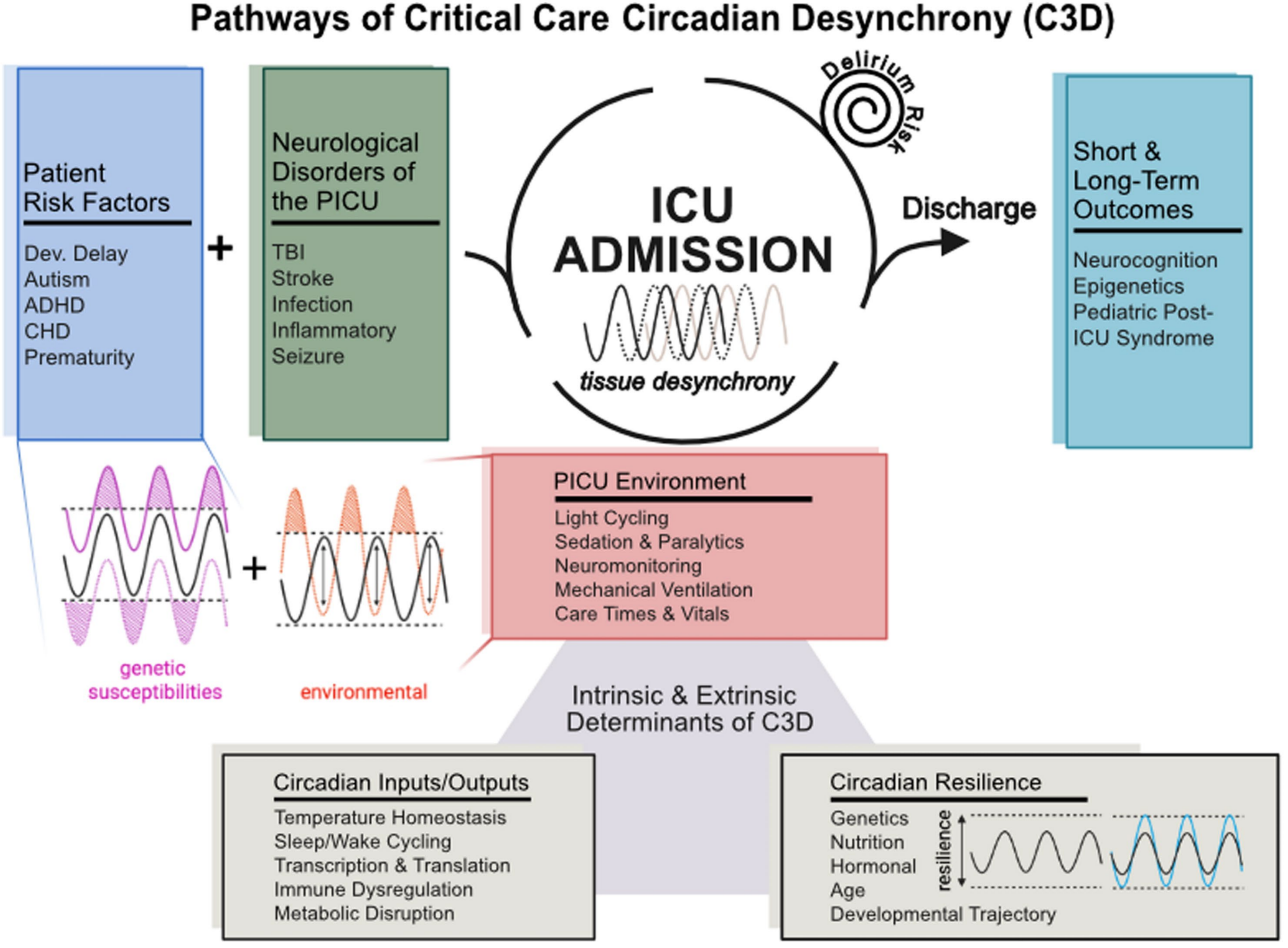
Outline for pathways of critical care circadian desynchrony (C3D). We note that the ICU setting introduces a concentration of risk factors from individual patients, underlying disease processes, and the environmental disruptions of critical care that can promote desynchronization of circadian rhythms. We hypothesize that different patients will exhibit measurable degrees of resilience to circadian perturbation depending on the combination of these risks. We predict that mitigation of C3D would decrease the risk of delirium and reduce time to discharge from the ICU.

The critical care environment can be conceptualized as a powerful circadian perturbation (Figure 3). It simultaneously disrupts environmental zeitgebers, pharmacologic state, arousal architecture, feeding timing, temperature regulation, and clinician-patient interaction rhythms. This is particularly consequential in pediatric neurocritical care because the developing brain may be highly sensitive to timing of signals.

Light–dark cycle timing is a known zeitgeber (60). In the NICU and PICU, light exposure may be continuous, irregular, excessive at night, or insufficient during the day. Noise, touch, handling, rooming-in, procedures, alarms, and care timing may further alter infant and child behavior and sleep–wake states (61–66). Potential zeitgebers within the NICU, and likely the PICU as well, include feeding, breastmilk composition, noise, touch, rooming-in, care times, and procedural timing (65, 66).

For example, sleep and circadian dysregulation in the PICU is exacerbated by the many interventions required for the infant with HIE including frequent cares, EEG, seizure management, and in some cases, therapeutic hypothermia. The physical environment, including noise and light, can directly alter infant behavior and sleep–wake states (61, 62). Further, prematurity is thought to predispose an infant to a lack of resilience to these rhythm-altering inputs (63). Likewise, iatrogenic interventions, including painful stimuli and handling, may cause further dysregulation (64). The impact of caffeine, which is commonly used in cases of neonatal apnea, remains relatively unclear but may impact wakeful state and active sleep (37).

Sedation and analgesia are central to pediatric critical care and have major effects on sleep and circadian organization. Pediatric patients may receive dexmedetomidine, benzodiazepines, opioids, ketamine, propofol, and other agents (67). Benzodiazepines are independent risk factors for delirium in the PICU (68, 69). Dexmedetomidine has been favored in some settings because pediatric and adult studies suggest associations with reduced benzodiazepine and opioid use, reduced mechanical ventilation duration, generally preserved sleep architecture with increased N2 sleep, and reduced delirium rates (69–76). However, many PICU patients receive multiple sedation modalities, and in a prospective cohort of mechanically ventilated PICU patients receiving combinations of opioids, dexmedetomidine, and intermittent benzodiazepines, sleep remained fragmented, diurnal variation was absent, 75% demonstrated no REM sleep, and average sleep periods lasted only 25 min (77). Higher doses of ketamine and propofol further disrupted sleep. In that cohort, dexmedetomidine did not improve sleep (77). These findings support an approach that emphasizes reduction in total sedation time and careful attention to sedative choice to reduce adverse effects on sleep and circadian rhythms (69).

Therapeutic hypothermia, fever, altered temperature variability, and autonomic instability represent additional interfaces between ICU care and circadian physiology (78, 79). In neonatal hypoxic–ischemic encephalopathy, therapeutic hypothermia intersects directly with thermoregulation, metabolism, sleep-state emergence, and secondary injury. More broadly, temperature and blood pressure rhythms may function as both measurable outputs and mechanistic contributors to recovery or injury progression.

The circadian biology of clinicians and practitioners may also influence care. Shift work and time of day affect circadian rhythms, inflammatory responses, fatigue, cognitive function, and gene expression profiles in healthcare workers (66, 80–82). Thus, the ICU is not only a circadian-disruptive environment for patients; it is also a setting in which caregiver circadian disruption may influence decision-making, medication timing, procedure timing, and care quality.

Approaches to managing these influences include circadian rhythm-focused ICU bundles and pharmacologic administration of melatonin (83, 84). However, intervention studies in pediatric neurocritical care remain limited, and the field lacks standardized developmentally appropriate measurement strategies and disease-specific circadian phenotypes.

## Circadian interfaces with common neurological disorders in the pediatric ICU

Pediatric neurocritical care encompasses critically ill children with a wide range of brain disease. The conditions below are common or clinical important presentations for pediatric neurocritical care specialists and intensivists. They are considered through a shared framework: sleep–wake disruption, alternation of circadian outputs, injury-induced clock disruption, ICU-related desynchronization, and opportunities for measurement or intervention.

### Hypoxic–ischemic encephalopathy

Hypoxic–ischemic encephalopathy (HIE) in the newborn refers to the central nervous system dysfunction related to a hypoxic–ischemic injury and serves as a prototypical pediatric neurological disorder highlighting the close interplay between the ICU environment, neurological dysfunction, and circadian rhythm dysregulation (85, 86). HIE is among the most common diagnoses encountered by neurologists in the neonatal ICU (estimated 38% of cases) (87) and one of the most common causes of acquired neonatal brain injury (88).

HIE results from global hypoxia-ischemia that can occur during a sentinel perinatal event, such as umbilical cord prolapse, placental abruption, or uterine rupture. Clinical features raising suspicion for HIE include significant alterations in fetal heart rhythms, reduced Apgar scores, and acidosis on fetal umbilical artery testing (86). Injury occurs in a multi-phasic sequence, involving primary energy failure followed by delayed secondary injury associated with reperfusion, oxidative injury, edema, and disrupted autoregulation (89). HIE can have lifelong ramifications, including worse cognitive, language, and motor outcomes (90, 91).

HIE is associated with significant dysregulation of circadian outputs, especially sleep–wake cycling. Delayed onset of cycling is often observed (92). In severe cases, the absence of developing sleep–wake cycling is associated with worse outcomes as the presence of cycling reflects proper functioning of cholinergic pathways (93). Prognostication studies in infants undergoing therapeutic hypothermia have revealed that infants who do not develop sleep–wake cycling by 36–45 h after birth tend to have worse outcomes (94). Earlier onset sleep–wake cycling beginning earlier and better sleep quality are associated with reduced death and fewer abnormal neuroimaging findings (95).

Sleep–wake dysregulation in HIE is linked to other circadian outputs: improved sleep quality in infants undergoing therapeutic hypothermia is also associated with reduced IL-1β and erythropoietin levels, whereas worse sleep quality is associated with elevated levels of TNFα, suggesting immune activation may influence altered cycling. Metabolic rhythmicity can also be disrupted in HIE, with altered cerebral glucose metabolism reported in HIE (96). Human genetic studies remain sparse, but data from rats suggest that hypoxic–ischemic injury can result in delayed alterations in *CLOCK* and *BMAL1* mRNA levels about 36–48 h after injury, coinciding with the secondary phase of injury (97).

### Traumatic brain injury

Traumatic brain injury (TBI) is the most common neurological disorder treated in the PICU (1). TBI may result from a variety of different types of external forces exerting a direct or indirect impact on the brain, ultimately resulting in alteration in brain function (98). This can include changes in level of consciousness, anterograde or retrograde amnesia, neurological deficits, or other alterations in mental status (99). Long-term ramifications of TBI can include persistent post-concussive symptoms and, in severe cases with repetitive TBI, chronic traumatic encephalopathy with features of cognitive or behavioral changes and pyramidal, parkinsonian, or cerebellar symptoms (100). While this latter disorder is seen more exclusively in adult populations, the onset of recurrent TBI preceding development of the disease can occur during childhood.

Sleep–wake dysregulation is a well-established phenomenon in pediatric TBI (99), with 53% of individuals reporting sleep–wake disorders in a recent pediatric prospective cohort (101). This dysregulation within the TBI cohort included disruption in initiation and maintenance of sleep (43%), sleep breathing disorders (5%), disorders of arousal (5%), sleep–wake transition disorders (25%), hypersomnolence (20%), and sleep hyperhidrosis (8%) (101). In another single-institution, retrospective cohort study (101) that examined TBI of varying severities, 68% had clinically relevant sleep–wake disorders, including disorders of initiating and maintaining sleep (53%), sleep breathing disorders (11%), disorders of arousal (15%), sleep–wake transition disorders (31%), disorders of excessive somnolence (10%), and sleep hyperhidrosis (11%). 43% required prescription of a new sleep treatment (102). In this latter study, sleep–wake disorders were significantly associated with pre-admission neurodevelopmental comorbidities and with worse global executive composite scores. Lack of sleep architecture in continuous EEG studies has been associated with poor neurocognitive and functional outcomes (103) and, complementing this in a separate study (104), normal sleep features on EEG were associated with favorable outcomes.

Beyond sleep–wake disruption, TBI is associated with altered blood pressure, temperature, hormonal regulation and melatonin secretion (105–108). Adult TBI studies have demonstrated reduced melatonin secretion, whereas pediatric TBI has been associated with increased peak melatonin – the mechanism underlying this discrepancy remains unclear (107, 108) Rat models show dysregulated *Bmal1* and *Cry1* expression after TBI rat models, suggesting that brain injury may directly affect the circadian clock directly (109). In adults with TBI, impaired circadian rhythms of brain temperature were associated with poorer outcomes suggesting that maintenance of circadian timekeeping could be a prognostic biomarker in future studies (110).

Another area of investigation is the interplay of the glymphatic system, TBI, and sleep dysregulation (111). Toxic cellular products released after TBI are suspected to cause dysregulation of the glymphatic system, which is thought to be predominantly active during sleep (112). Sleep dysregulation may then further impair toxin clearance, creating a feed-forward cycle. While therapies directly targeting glymphatic dysfunction remain undefined, proposed circadian-oriented interventions include melatonin, blue light therapy, treatment of sleep-disordered breathing, and nutritional supplements such as omega oils (111). Pediatric TBI therefore illustrates how circadian disruption may influence recovery through sleep architecture, hormonal timing, temperature rhythms, glymphatic clearance, and injury-induced clock disruption.

### Stroke

While pediatric stroke can be ischemic or hemorrhagic, its causes, treatment, and clinical course differ from adult cerebrovascular disease. Arterial ischemic stroke and sinovenous thrombosis constitute the major forms of ischemic stroke seen in children; hemorrhagic stroke may occur within the brain parenchyma, intraventricular space, or subarachnoid space (113). The overall incidence of stroke in children is estimated to be around 1.3–13 strokes per 100,000 children and comprises roughly 10% of neurological disorders seen in the PICU (1, 113). Pediatric stroke differs from adult stroke in mechanisms of vascular disease, collateral formation, neuroplasticity, inflammatory contribution, and recovery potential.

The causes of pediatric stroke are varied, and approximately half are cryptogenic (114). Risk factors include infection, congenital heart disease, inborn errors of metabolism, mitochondrial disorders, genetic disorders, hematologic disease, rheumatologic disorders, congenital cerebrovascular abnormalities, arteriovenous malformations, and idiopathic cerebrovasculopathies such as Moyamoya syndrome (115).

Adult ischemic and hemorrhagic stroke are associated with significant changes in global circadian organization (116, 117). Infarct size, progression, and expression of circadian clock-related genes may be influenced by whether an ischemic stroke occurs during the daytime or nighttime (116, 118). Pediatric data are more limited, but a prospective pediatric stroke cohort found that 64% of patients had sleep–wake disorders (101). In adults, ischemic stroke can result in loss of circadian rhythmicity in autonomic systems such as blood pressure (119) and dysregulation of temperature and other rhythms is associated with worsened outcomes as well (120).

Time-of-day pattens in stroke may differ between children and adults. Adult stroke has been reported to peak in the overnight hours or in the morning depending on cohort and methodology (121–123). In contrast, a multi-institutional study of a cohort of 478 pediatric patients with acute ischemic stroke revealed that most strokes occurred in the afternoon (124) with nocturnal strokes more commonly associated with arteriopathic risk factors. This difference may reflect developmental variation in circadian rhythms, distinct pediatric stroke mechanisms or infection- inflammation-related arteriopathy rather than atherosclerotic mechanisms common in adults (113).

Circadian immune regulation may influence stroke risk and recovery. Experimental ischemia activates inflammatory pathways involving IL-6, TNFα and inflammatory cells, many of which vary with circadian time (125, 126). Mouse models have demonstrated that the degree of immune response to an ischemic event can be influenced by circadian time (127). BMAL1 regulates interleukin expression in microglia and neutrophil activity, providing plausible molecular links between the clock and post-stroke inflammation (128). Hypoxia responses and angiogenesis also vary with circadian time. PER2 and BMAL1 interact with HIF-1α, and VEGF1 rhythmicity may be regulated by BMAL1 (129). Circadian regulation of blood–brain-barrier permeability may influence inflammatory signaling and xenobiotic transport (130, 131, 132).

Hemorrhagic stroke remains a growing area of interest as well. Intracranial hemorrhage may lead to a variety of deleterious effects. The primary insult can involve reduced perfusion to certain regions of the brain as well as local mass effect from the enlarging hematoma. Secondary injury can be due to oxidative stress and innate hemoglobin lysate, which induces ferroptosis, a form of regulated cell death (133). Murine models of acute kidney injury suggest that direct targeting and inhibition of *Rev-erbα*, another circadian clock component that has been reported as a heme ligand (134), reduces severity of ferroptosis (135). Whether such a methodology would potentially reduce the severity of injury in intracranial hemorrhage is unknown (136). Akin to the impact of ischemic stroke, the injury from intracranial hemorrhage is likely to be subject to circadian variability. A murine model of subarachnoid hemorrhage found that cerebrovascular myogenic tone is rhythmic and that the timing of injury during the circadian cycle directly impacts the degree of injury (137). Similarly, in an isolated retrospective study of three adult cases of subarachnoid hemorrhage, increased temperature variability correlated with elevations in intracranial pressure the following day suggesting a direct impact on circadian outputs (78).

### Infectious and inflammatory diseases

The immune system plays a critical role in both infectious and inflammatory diseases. Sleep disruption can have a direct, suppressive effect on the immune system (138, 139). Similarly, different cytokines have distinct effects on sleep: Interleukin (IL)-1, TNF-α, and IFN-*γ* have been shown to enhance sleep, whereas TGF-β, IL-10, and IL-13 are thought to inhibit sleep (140). Neuroinflammation and circadian rhythms have been extensively reviewed elsewhere (53, 141, 142).

Sepsis is a multi-system disorder with that highlights the interplay with circadian biology (143–145). Reduced or disrupted rhythmicity in circadian clock genes, melatonin, cortisol, and temperature in adults with sepsis are correlated with increased disease severity and in some cases length of stay (146–149). These disrupted rhythms are seen in the immune cells themselves, as downregulation of clock gene expression occurs in monocytes acquired from septic patients (150). Temperature amplitude and frequency patterns have been associated with the development of sepsis in trauma patients, and the pattern of temperature fluctuations can be predictive of mortality (151, 152). Blood pressure variability has also been examined as a prognostic feature in sepsis (153). Pediatric data are more limited but circadian variation in plasminogen-activator-inhibitor-1 (a protein that plays a role in coagulation mechanics) is altered in pediatric meningococcal sepsis (154).

Encephalitis is a frequently encountered neuroinflammatory disorder within the PICU (155). Encephalitis may result from direct infection, a post-infectious inflammatory response, or an underlying autoimmune etiology. Commonly seen causes of infectious encephalitis within the PICU include West Nile virus, herpes simplex virus, and enterovirus (156). The most common cause of autoimmune encephalitis in pediatric populations is anti-N-methyl-D-aspartate receptor (NMDAR) encephalitis (156, 157). In NMDAR encephalitis, autoantibodies targeted against the NMDAR lead to a striking immune response in the brain that can lead to progressive psychiatric symptoms, seizures, movement disorders, and even coma (158).

While we could not identify any studies examining its impact on intrinsic circadian physiology, autoimmune encephalitis has been associated with sleep disorders in both pediatric and adult populations (159–161). NMDAR encephalitis can uniquely present with an epoch of severe insomnia, followed by episodes of psychomotor irritability or hypersomnia during recovery (149, 162). One retrospective cohort of pediatric NMDAR encephalitis cases estimated that 95% have sleep problems at onset and 27% continue to have sleep problems at 1 year after diagnosis (163). Increased confusional arousals in NREM sleep are also reported in pediatric populations and case reports in adults suggest that both REM and NREM sleep parasomnias can develop (164, 165). A recent study by Liu and colleagues has proposed that this sleep dysregulation in autoimmune encephalitis may be related to a dysfunctional metabolic network, including alterations in homocysteine and neuron-specific enolase, although the exact mechanism and whether these are byproducts of another underlying process still remains elusive (166). Ultimately, recognition of sleep dysregulation may serve a role in earlier detection of autoimmune encephalitis (167). Importantly, autoimmune encephalitis treatment often involves administration of corticosteroids, which can have a direct impact on sleep–wake dynamics as well (168, 169). Altogether, while much remains to be learned, NMDAR encephalitis likely plays a distinct role in dysregulation of circadian rhythms that manifests through the output of sleep.

Infectious encephalitis is less well studied in pediatric circadian contexts. Tick-borne encephalitis has been associated with circadian rhythm and sleep dysregulation in pediatric cohorts (169). In adults, Epstein–Barr virus (EBV)-related encephalitis has been associated with circadian and sleep dysregulation (170). Interestingly, a single-center cohort study examined sleep dysregulation, among many other clinical features, seen in a variety of causes of pediatric encephalitis, including enterovirus, *Mycoplasma pneumoniae*, HSV, and NMDAR (171). These differences suggest that sleep and circadian phenotypes may vary substantially across autoimmune and infectious etiologies, perhaps reflecting distinct inflammatory, metabolic, or network-level mechanisms.

### Epilepsy and status epilepticus

The treatment of seizures is one of the primary challenges of the pediatric neurologist in the intensive care setting. Time-of-day specificity of seizures has been noted for more than a century and a rapidly growing interest in characterizing potential rhythms of seizure incidence and the conversion to status epilepticus holds promise for using timing in therapeutic interventions. The relationship between seizures, sleep, and circadian rhythms has been thoroughly reviewed (172, 173) but few studies have directly examined how the ICU circadian desynchrony affects seizure control.

In human subjects monitored for years with intracranial detection tools, both seizures and inter-ictal electrical activity (IEA) demonstrate patterns that in some individuals are durably circadian and in others multidien (rhythm of several days); in some individuals phase alignment between both timing scales may maximize seizure burden (174). In many cases and in most studies, it remains unclear whether time-of-day seizure incidence is directly mediated via a circadian clock or is secondary to associated behavioral states such as the vigilance or sleep state because most reports have not examined the timing of seizure incidence or IEA with concomitant measurements of endogenous circadian phase.

Temporal patterns in epilepsy have been primarily characterized in adults, although certain classical clinical epilepsy syndromes such as juvenile myoclonic epilepsy have strong predilection for onset of seizures in the morning. In a recent report, focal epilepsies were found to have a circadian pattern in 89% of adult study participants (175). Molecular mechanisms linking the circadian timekeeper to temporal patterns in epilepsy still remain an area of active research; however, we can look to both human and animal studies for hints. Disruption of core clock proteins BMAL1, CLOCK, PAR/ZIP, and REV-ERBα transcription factors have been associated with epilepsy (176–181). CLOCK was found to be suppressed in focal cortical dysplasia; its reduction in mouse models precipitated hyperexcitability and sleep-related seizures (182). Presumed loss-of-function mutations in *BMAL1* in association with a neurodevelopmental syndrome that includes some individuals with epilepsy, again suggests a link between the core circadian clock and seizures risk (183).

Because approximately 70% of the synaptic transcriptome is under clock control (21), the relationship between clock protein and epilepsy is plausible but complex. *BMAL1* loss ablates circadian function but also affects the expression of many non-rhythmic genes. Indeed, BMAL1 (and other clock proteins) have biochemical functions in the cell beyond transcription. For example, BMAL1 is rhythmically localized to synapses in response to its phosphorylation. In synapses, BMAL1 regulates the circadian organization of synaptic biochemistry (28). The impact of this finding on epilepsy remains to be determined. Circadian oscillation of intracellular chloride in the cortex at a concentration near the reversal potential for GABA suggests another mechanism by which circadian rhythms could regulate excitability (24).

Genes associated with epilepsy have a strong role in the regulation of circadian timekeeping. SCN1A mutations cause Dravet syndrome, Genetic epilepsy with febrile seizure plus (GEFS+), and early onset development and epileptic encephalopathy (DEE). Importantly, mutation of SCN1 has a marked effect on the circadian cycling of the suprachiasmatic nucleus (184, 185). Similarly, pediatric refractory status epilepticus may peak in the morning, suggesting a diurnal nature of clinical presentation (186).

We could identify no studies that directly examined the interplay of circadian rhythms and epilepsy in the PICU setting. We might predict that the relationship is bidirectional such that the disruption of circadian rhythms in the ICU (Figure 3), might precipitate disruption of control of neuronal excitability, sleep/wake cycles, hormonal control, inflammatory responses, and drug metabolism that could contribute to lowered thresholds for seizures. Return of sleep cycling has been associated with favorable outcome in refractory status epilepticus, emphasizing the need for pediatric neurocritical studies that integrate seizure burden, sleep state, circadian phase, medication timing, and ICU environment.

### Delirium and disorders of consciousness

Consciousness can be divided into content and arousal (187). Content reflects an awareness or attention toward oneself and the surrounding environment. Delirium is a waxing and waning disruption of attention or awareness that develops over a short timeframe and is a clear deviation from an individual’s baseline (188). Delirium occurs in up to 80% of patients in the PICU according to the most recent Society for Critical Care Medicine guidelines. Delirium is associated with increased length of stay, duration of mechanical ventilation, mortality, and increased hospital costs (68, 69). Pediatric delirium may be hypoactive, hyperactive, or mixed; hypoactive delirium is most common. Risk factors include neurodevelopmental delays, younger age, poor nutritional status, and cyanotic heart disease (69).

Delirium is associated with disruptions in sleep and circadian rhythms (189, 190). Its onset and persistence are multifactorial, involving sedative exposure, type of sedation, underlying clinical context for admission, irregular feeding, sleep/wake disruption, disruptive lighting, noise and the ICU care environment. The relationship is likely bidirectional: delirium disrupts circadian rhythms and sleep/wake patterns, which further exacerbates the alignment between internal clocks and the environment, slowing recovery from delirium (68). Whether disruption of circadian clocks is the primary inciting element in the onset of delirium is unknown and, in most cases, difficult to ascertain. This feed-forward cycle underlies the diurnal dysregulation hypothesis of delirium, although neuroendocrine and neuroinflammatory mechanisms are likely influencing factors (68, 191).

Recent PICU clinical practice guidelines emphasize routine screening for delirium, minimizing overall sedation exposure, minimizing benzodiazepine exposure, sleep hygiene optimization, interdisciplinary rounds, and family engagement and involvement in care (69). Sleep hygiene protocols and interventions commonly include noise reduction, spaced care times, and timed lighting (69, 192). Whether these interventions directly reduce the incidence of delirium in the PICU remains uncertain (192–194). Delirium should therefore be considered a transdiagnostic circadian-relevant syndrome in pediatric neurocritical care: both a consequence of circadian misalignment and a potential amplifier of further desynchrony.

### Other diseases relevant to the PICU

Here we address status dystonicus and non-neonatal global hypoxic–ischemic as these are frequently seen in the PICU. Dystonia is a hyperkinetic movement disorder that involves sustained or intermittent involuntary muscle contractions that can result in abnormal posturing or repetitive movements (195). Status dystonicus reflects the most severe form of sustained or unabating dystonia and constitutes a medical emergency because it can compromise respiratory and bulbar function, cause severe pain and produce metabolic derangements (196). While status dystonicus is more commonly seen in the pediatric setting, increasing prevalence is also observed in adults (197). In our review of the literature, there are no devoted studies investigating the impact of status dystonicus on sleep and circadian rhythm dysregulation within the PICU. However, impaired sleep is one of the first recognizable signs in the onset of status dystonicus and is an integral component of a more recently proposed dystonia severity scale (196, 198). Promotion of sleep through use of sedating medications, such as dexmedetomidine and use of a sleep–wake dystonia diary is foundational to management (196). This is an area ripe for further investigation into the interplay between the disorder itself and intrinsic circadian rhythms.

Non-neonatal global hypoxic–ischemic injury is a form of acquired brain injury that can occur in the setting of cardiac arrest. In a prospective cohort of pediatric patients admitted to the PICU, 33% of individuals with acquired hypoxic ischemic injury had sleep–wake disorders, with the most common disorder involving initiation and maintenance of sleep (101). Limited evidence from pediatric anoxic coma suggests that major alteration in sleep architecture can occur (199). Sleep features also carry prognostic value: early sleep spindles within 24 h of arrest have been associated with improved outcomes (200). Because spindles reflect preservation of widespread thalamocortical networks, their absence may indicate severe network dysfunction (201). Further investigation is needed to understand the prognostic and mechanistic value of sleep architecture and circadian biomarkers in pediatric hypoxic–ischemic injury beyond the neonatal period.

## Measuring rhythms in pediatric neurocritical illness

A variety of tools and metrics for measuring sleep have been implemented within the critical care setting. These range from patient or caregiver reported surveys to the gold standard of polysomnography (PSG). PSG synthesizes cortical activity, muscle activity, cardiac activity, respirations, and oximetry to provide a comprehensive evaluation of an individual’s sleep state (202). This involves utilization of a combination of EEG, electromyography, electro-oculogram, electrocardiogram, nasal and oral air flow, nasal pressure transducers, and pulse oximetry. Diagnostic indications for PSG in pediatric populations include evaluation for obstructive sleep apnea, congenital central alveolar hypoventilation syndrome, sleep-related hypoventilation due to neuromuscular disorders, or sleep-related hypoventilation due to chest wall deformities (203). There are no formal guidelines that we identified on the use of PSG in the PICU. Polysomnography can provide direct information on sleep architecture; however, must be balanced with technical requirements (placing the leads, electrode maintenance, skilled interpretation) and are prone to atypical features that may increase the difficulty in accurate interpretation of the current sleep state (204).

Continuous EEG monitoring within the PICU largely focuses on the detection of seizures, evaluation for non-convulsive status epilepticus, and prognostication after brain injury (205). While not yet well-validated within the ICU, continuous EEG with use of a full electrode montage may also adequately capture sleep states (206, 207). Quantitative EEG, which performs high-level analysis of raw EEG data and trends utilizing Fourier transformation, in isolation may be sufficient for sleep staging. A recent investigation using density spectral array, a form of quantitative EEG, for sleep stage interpretation by EEG as compared to gold standard PSG revealed relatively good agreement in the classification of wake and sleep states, although this agreement decreased with finer detail categorization of NREM states (207). Despite these current limitations, direct evaluation of changes in diurnal patterns and other markers of circadian rhythms may still be feasible.

Actigraphy uses a wrist or ankle device to assess rest-activity rhythms. It is relatively straightforward and low cost but depends on movement changes to infer sleep and wake. This limits accuracy in sedated, immobilized, mechanically ventilated and some neurologically impaired children. Pediatrics studies have shown incongruence between activity and PSG in some critical care contexts (13, 208).

Patient and caregiver-reported surveys provide additional information but are vulnerable to bias and perceptual inaccuracies (13, 204). Biomarker-based approaches include melatonin, urinary melatonin byproducts, cortisol, temperature, cytokines, and metabolic markers like glucose, NAD/NADH ratio. Several groups have developed gene batteries that can report circadian phase from a single measurement (13, 107, 209–211).

The critical care setting also contains abundant continuously collected data that may encode circadian information, paving the way for advances in multimodal neuromonitoring (212). Such an approach utilizes a combination of strategies to evaluate cerebral function, with the primary goal of identifying individuals who are at high risk of primary or secondary neurological injury. In addition to EEG, pupillometry, continuous vital sign monitoring, and near-infrared monitoring have been utilized. Additional methodologies, such as intracranial pressure monitoring, ventilator parameters, medication timing, sleep timing, nursing care times and environmental light or noise exposure could also help expect the field of invasive and noninvasive circadian rhythm monitoring. Early advances include associations between temperature variability and elevated intracranial pressure the following data, as well as modeling of the circadian patterns using pulmometry in subarachnoid hemorrhage (78, 213). We could not identify any publications specifically using multimodal neuromonitoring to evaluate circadian rhythms within the PICU; however, such a complementary approach may be helpful in measuring changes in circadian rhythms for those highly susceptible to neurological injury (214).

Future measurement strategies must distinguish sleep disruption, arousal-state disruption, homeostatic sleep pressure, intrinsic circadian phase and peripheral clock desynchrony. Multimodal approaches integrating EEG, biomarkers, actigraphy, continuous monitoring, environmental exposure, medication timing, feeding schedule, and clinical outcomes will likely be necessary.

## Chronotype, developmental timing, and personalized circadian risk

Chronotype reflects individualized circadian entrainment set-points influenced by genetics, disease state, geography, culture and environment (215). Characterization of chronotype may supported personalized medicine by opening opportunities for chronotype-specific treatment approaches. Although chronotypes may manifest in early childhood, their application to critical care has mostly been studied in relation to provider shift work rather than patient-specific pediatric illness. Additionally, it is likely that an individual’s specific chronotype will change over time and throughout their developmental progression.

In pediatric neurocritical care, a patient’s specific chronotype would be informed by their developmental stage, neurological diagnosis, acute injury state, ICU environment, medication exposures, feeding timing, and sleep–wake architecture. A child’s apparent rhythm in the PICU may reflect intrinsic chronotype, developmental physiology disease-induced dysrhythmia, medication effects, environmental desynchronization or a combination thereof. Future work should therefore define developmentally appropriate and disease-specific norms before chronotype can be used clinically in NCCC.

## Emerging interventions and chronotherapeutic opportunities

Circadian medicine aims to use temporal organization of biomarkers, drug targets, drug metabolism, and the rhythms of health care implementation to optimize diagnosis, care, and outcomes (15). Its application in pediatric medicine is still nascent. Nevertheless, several intervention domains are emerging.

Environmental entrainment strategies include timed light exposure, preservation of day-night structure, noise reduction, care clustering, family engagement, procedural timing, and minimizing unnecessary nighttime disruptions. These strategies are conceptually appealing because they address the effect of zeitgebers upstream and can therefore have distributed impact on the whole circadian system. ICU circadian ‘bundles’ that package multiple environmental elements into systematic interventions have been proposed in both adult and neonatal settings (83).

Sedation-aware circadian care is another major opportunity. Sedatives and analgesics have profound influences on sleep architecture, arousal, delirium, and respiratory support. It is therefore imperative that circadian-supportive care should include reduction of total sedation burden, benzodiazepine minimization, and attention to the risk/benefit ratio of a sedative’s potency compared with its sleep and circadian effects. For example, dexmedetomidine may preserve sleep features, but existing pediatric evidence is mixed (70–77).

Melatonin is a commonly used chronobiotic in both adults and pediatric critical care (209, 216). Other pharmacologic approaches to the intrinsic clock target casein kinase, REV-ERBs, RORs, and CRYs (217). However, pediatric neurocritical care trials are needed before these strategies can be formally recommended. Therapeutic pharmacologic manipulations of the clock will likely be disease-, age-, and phase-dependent.

Chrono-nutrition is another important domain. Abundant evidence from animal models suggests that circadian rhythms are strongly entrained by timed feeding regimens. Desynchronizing food availability from rest/activity cycles promotes metabolic derangement; conversely, enforcing timed feeding in animals that have biologically disrupted circadian clocks establishes behavioral rhythms and has long-term benefit in ameliorated glucose tolerance and hepatic dysfunction (218–220). Therefore, it is logical to expect that disrupted feeding patterns that frequently accompany critical care management will have a strong disruptive effect on circadian coordination of metabolism. In adults, there is some evidence to suggest that time-restricted eating may improve metabolic health and the gut microbiome (217). While similar studies have yet to be extensively studied in pediatrics, the PEPaNIC trial demonstrated that delayed initiation of parenteral nutrition in the NICU is associated with reduced rates of infection, faster recovery, and better neurocognitive outcomes after two years (221–223). In fact, changes in DNA methylation were observed even two years afterward (224). Recent investigations with conflicting results have explored the potential impact of intermittent bolus feeding as compared to continuous feeding within the ICU, but whether this improves outcomes remains unclear and randomized trials for pediatric populations are in progress (225–227). Whether time-optimized nutrition improves circadian alignment or neurological outcomes in the setting of neurocritical care remains unknown.

Chronotherapy refers to the timing of treatments to match biological rhythms and may have applicability to antiseizure medications, corticosteroids, antihypertensives, immunotherapies, and other interventions relevant to pediatric neurocritical care. However, formal, well-controlled studies are needed. Areas of promise include T-cell therapies, chemotherapy, and seizure control. Ideas on the use of chronotherapies in the management of hypertension, cardiovascular disease, and epilepsy are also emerging (136, 228–230). Even the timing of chimeric antigen receptor T-cell therapy has been shown to be influenced by circadian time in observational studies (231).

We note that caution is needed because under certain conditions, or for specific disorders, circadian desynchrony may be *adaptive* rather than pathogenic. Thus, mechanistic studies, biomarkers, and prospective trials are needed.

## Toward a framework for pediatric circadian neurocritical care

A translational framework for pediatric circadian neurocritical care should begin with the identification of factors that place the circadian system at risk of misalignment: developmental state, neurological diagnosis, sedation exposure, light–dark disruption, feeding schedule, temperature manipulation, mechanism ventilation, delirium risk assessment, seizure burden, and baseline sleep–wake organization.

A natural next step is to standardize the methodologies used for assessment of circadian phase. Existing clinical data can be leveraged to characterize sleep architecture, melatonin and cortisol rhythms, temperature, periodicity of vital signs, rest-activity, pupillary rhythms, environment exposure, medication timing, and feeding schedules. Therefore, multimodal rhythm phenotyping may allow disease-specific circadian signatures to be defined.

Circadian-supportive care should aim to protect entrainment when safe and feasible. This may include light–dark re-structuring, reduction in noise and disruption, clustering of cares, preserving family presence, minimizing unnecessary sedation, consideration of feeding timing, and coordination of procedures when clinically feasible. While a natural progression of the prior steps, many of these are already being implemented in critical care guidelines (69).

We surmise that there may be disease-specific circadian endophenotypes: variants of disease that are particularly susceptible or resilient to circadian disruption or even, particular domains of circadian disruption. If future research bears this hypothesis out, then disease-specific circadian interventions should be studied. Outcomes should include mortality, length of stay, but also delirium presence and extent, seizure burden, inflammatory markers, metabolic stability, autonomic control, functional recovery, quality of life metrics, and most crucially, long-term neurodevelopment.

## Conclusion and future directions

Consideration of timing remains a relatively under-explored area of pediatric neurocritical care. Neurological injury evolves over hours to days and recovery unfolds over weeks or even years. The developing brain is embedded within a dynamic circadian system that organizes its daily structure in relation to chronological age and developmental trajectory. Circadian rhythms organize physiology across molecular, cellular, organ, systemic and behavioral scales. In the PICU and NICU, the entire landscape of these rhythms is multifariously challenged by neurological injury, environmental disruption, sedation, altered patterns of nutrition, temperature dysregulation and manipulation, mechanical ventilation and care-delivery rhythms.

Sleep and circadian rhythms dysregulation are reported across several pediatric neurocritical disorders, including neonatal hypoxic-ischemia encephalopathy, TBI, stroke, infection and inflammatory diseases, epilepsy, delirium, status dystonic, and non-neonatal hypoxic–ischemic injury. Considerable current evidence has been extrapolated from adult critical care of preclinical studies, but key differences in pediatric populations are already evident and likely reflect developmental variation in sleep–wake cycles, circadian consolidation, immune function, vascular biology, metabolism, underlying mechanisms of neuronal excitability, and brain recovery mechanism.

The field requires developmentally appropriate circadian biomarkers, disease-specific rhythm phenotyping, multimodal monitoring strategies, and prospective trials of circadian-supportive interventions. The stage is set for pediatric circadian medicine, but its implementation must be deliberate, mechanistically rooted, and developmentally informed. Integrating circadian biology into pediatric neurocritical care may improve biomarker development, prognostication, timing of therapeutics, environmental optimization, sedation strategies, nutritional care, and long-term neurological outcomes.
